# Transcranial Direct Current Stimulation in Post-stroke Chronic Aphasia: The Impact of Baseline Severity and Task Specificity in a Pilot Sample

**DOI:** 10.3389/fnhum.2017.00260

**Published:** 2017-05-29

**Authors:** Catherine Norise, Daniela Sacchetti, Roy Hamilton

**Affiliations:** Laboratory for Cognition and Neural Stimulation, Department of Neurology, University of PennsylvaniaPhiladelphia, PA, United States

**Keywords:** aphasia, baseline severity, stroke, tDCS, neurorehabilitation

## Abstract

Emerging evidence suggests that transcranial direct current stimulation (tDCS) can improve aspects of language production in persons with chronic non-fluent aphasia due to left hemisphere stroke. However, to date, studies exploring factors that predict response to tDCS in this or any patient population remain sparse, as are studies that investigate the specific aspects of language performance that are most responsive to stimulation. The current study explored factors that could predict recovery of language fluency and which aspects of language fluency could be expected to improve with the identified factor(s). We report nine patients who demonstrated deficits in fluency as assessed using the Cookie Theft picture description task of the Boston Diagnostic Aphasia Examination. In the treatment condition, subjects received a 2.0 mA current through 5 cm × 5 cm electrodes for 20 min at a site previously shown to elicit a patient-dependent optimal response to tDCS. They were then tested 2-weeks and 2-months after treatment. In the sham condition, a subset of these subjects were tested on the same protocol with sham instead of real tDCS. The current study assessed language fluency improvements in measures of production at the word-level and sentence level, grammatical accuracy, and lexical selection as a function of baseline aphasia severity. A more severe baseline language profile was associated with larger improvements in fluency at the word-level after real tDCS but not sham stimulation. These improvements were maintained at the 2-week follow-up. The results suggest that for at least some outcome measures, baseline severity may be an important factor in predicting the response to tDCS in patients with chronic non-fluent aphasia. Moving forward, the ability to identify patient factors that can predict response could help refine strategies for the administration of therapeutic tDCS, focusing attention on those patients most likely to benefit from stimulation.

## Introduction

With 80,000 new cases in the US each year and a total of 6.4 million affected individuals, aphasia—acquired loss of language ability—is one of the most common and debilitating post-stroke cognitive disorders (Wade et al., 1986; National Stroke Association, 2008; Kyrozis et al., 2009). Post-stroke aphasia typically arises from injury to the left (dominant) hemisphere, in a network of language-related regions that surround the Sylvian fissure. The degree to which individuals recover from aphasia is variable, and chronically persistent deficits are common (Mimura et al., 1998; Rosen et al., 2000; Heiss and Thiel, 2006; Saur et al., 2006). Unfortunately, the efficacy of behaviorally-based rehabilitation approaches has proven limited (Winhuisen et al., 2005). However, a growing body of encouraging evidence now suggests that non-invasive neuromodulation techniques such as transcranial direct current stimulation (tDCS) may have the capacity to improve aspects of language production in persons with chronic aphasia (Monti et al., 2008; Baker et al., 2010; Fiori et al., 2011; Fridriksson et al., 2011).

TDCS modulates brain activity by delivering a weak polarizing electrical current, which is believed to induce incremental shifts in the resting membrane potentials of neurons (Nitsche and Paulus, 2001). These shifts, while insufficient to depolarize neurons acutely, can result in changes in neuronal firing rates, which in turn are associated with measurable changes in cognition and behavior (Schlaug et al., 2009). Repeated sessions of tDCS paired with a behavioral task have been associated with enduring changes in both neural activity and performance (Boggio et al., 2007; Reis et al., 2009; Brunoni et al., 2012) which has given rise to considerable interest in the use of tDCS as an adjunctive treatment in patients with post-stroke deficits, including hemiparesis (Peters et al., 2016), neglect (Yi et al., 2016), and aphasia (Monti et al., 2008; Baker et al., 2010; Fiori et al., 2011; Fridriksson et al., 2011; Medina et al., 2012).

To date, at least 19 papers have been published employing tDCS as a potential treatment for post-stroke aphasia (Cappon et al., 2016). Most of these studies have focused on patients with non-fluent aphasia, that is deficits primarily in language production. While non-fluent aphasia manifests itself in a variety of symptoms, including but not limited to slow effortful speech and agrammatism, the majority of tDCS studies in the field have focused on picture naming (Monti et al., 2008; Baker et al., 2010; Fiori et al., 2011; Flöel et al., 2011; Fridriksson et al., 2011; Richardson et al., 2015; Wu et al., 2015). There are both theoretical and practical reasons for this; difficulty with naming is a ubiquitous property of all conventional post-stroke aphasia syndromes, and it is one of the most straightforward language abilities to evaluate experimentally. However, while studies of the effect of tDCS on naming ability undoubtedly provide some insight into the utility of tDCS as a language intervention, these studies fall short in determining whether tDCS is likely to be helpful in restoring the ability to generate fluid, spontaneous, self-directed speech to patients who have lost this capacity.

In a previous work, we reported improvement of language abilities in a cohort of patients with chronic non-fluent aphasia 2 weeks and 2 months after a course of tDCS (2 mA × 20 min for 10 days; Shah-Basak et al., 2015). Depending on the results of an individual montage-testing phase, treatment was delivered on a subject-by-subject basis to either the left frontal lobe (targeting perilesional areas of the language dominant hemisphere) or the right frontal lobe (targeting presumed homologs of damaged left hemisphere language areas) using either anodal or cathodal tDCS. Compared to sham stimulation, patients showed significant and sustained improvement on the Western Aphasia Battery Aphasia Quotient (WAB-AQ), a global measure of aphasia severity. In the current study, we further explored the data obtained from these patients, in an attempt to determine whether and in what ways tDCS affected speech fluency. Our approach to examining fluency changes was informed by a prior investigation in which we employed quantitative production analysis (QPA; Saffran et al., 1989) to explore changes in spontaneous speech in chronic non-fluent patients who had received repetitive transcranial magnetic stimulation (rTMS), a different form of non-invasive neuromodulation (Medina et al., 2012). In that investigation, we explored changes in spontaneous speech at the level of word production, sentence generation, grammar, or lexical selection (speech efficiency), and found that subjects who had received TMS experienced an improvement in fluency that was largely due to increased production at the word level. Based on these prior findings, in this study we hypothesized that any improvement in speech fluency that was identified following tDCS would likely be most notable at the word level, rather than the level of sentences or overall narrative.

In addition to characterizing the specific language abilities that are likely to be affected by tDCS in patents with aphasia, it is important for investigators of begin to determine the clinical properties of patients that predict response to stimulation. One clinical characteristic that we argue should be considered is baseline symptom severity. Although clinical studies with tDCS have not yet fully explored the impact of baseline performance on tDCS-induced recovery, a few recent studies in healthy subjects have suggested that individuals who demonstrate weaker performance at baseline may be more likely to benefit from stimulation. For instance, Sarkar et al. (2014) enrolled healthy subjects to undergo a mathematical training task paired with tDCS. The investigators also measured subjects' mathematics anxiety, which is generally negatively correlated with mathematical proficiency. They found that subjects who were worse at mathematics and had high mathematics anxiety at baseline experienced a significant increase in math performance after tDCS to the dorsolateral prefrontal cortex, while those with strong baseline mathematical ability worsened after tDCS. In the language domain, Turkeltaub et al. (2012) found that, in healthy adults who underwent a single session of tDCS over the left posterior temporal cortex, reading efficiency improved more robustly in subjects whose baseline performance was below the mean level of performance of the study cohort. Both studies suggest that poor initial performance on cognitive tasks may predict greater tDCS-induced improvement. Moreover, an association between poor baseline functioning and a greater post-stimulation improvement has also been shown in non-cognitive tasks like motor coordination (Uehara et al., 2015). The same effect has been observed in studies of fine motor control that compared non-musicians to professional musicians (Furuya et al., 2014). Taken together, one interpretation of these studies is that brain networks associated with relatively weak performance on tasks may be more amenable to beneficial modulation via tDCS, while those associated with strong performance may already be closer to their optimal state and may thus benefit less—or may even be adversely affected—by further modulation.

However, while a small but growing body of evidence in healthy subjects suggests an association between weaker baseline performance and greater improvement after tDCS, it not yet been determined whether this relationship also pertains to the application of tDCS in persons with neurologic deficits, such as patients with post-stroke aphasia. At least two very different scenarios seem plausible. One possibility is that, as previous studies in healthy subjects have suggested, persons who perform poorly at baseline have language networks that can be improved further by tDCS, whereas patients who perform well at baseline may have language networks that are closer to an optimal state, and thus less likely to be enhanced by additional neuromodulation. However, it is also possible that patients who perform poorly at baseline have language networks that are so severely degraded by stroke that they cannot be enhanced substantively by tDCS, while better baseline language function may signal more robust residual language networks, which can be modulated beneficially by stimulation. This would be consistent with studies of the natural history of aphasia recovery, which suggest that patients who exhibit poorer language recovery early in their post-stroke course are less likely to have substantive improvement in aphasia severity compared to patients with less severe initial symptoms (e.g., Laska et al., 2001).

In the current study we sought to address two questions. First, using QPA (Gordon, 2006) we sought to determine whether there are the distinct elements of production within spontaneous speech that are preferentially affected by tDCS in patients with chronic non-fluent aphasia after stroke. We used the Cookie Theft narrative picture description from the Boston Diagnostic Aphasia Examination (BDAE) to assess language fluency. Based on our prior findings in a related population receiving TMS, we hypothesized that the most robust effects of tDCS on spontaneous speech would be in word level production. Secondly, we examined relationships between baseline severity on measures of speech fluency and response to tDCS in our cohort of patients with chronic aphasia. In light of the evidence in healthy subjects discussed above, we hypothesized that weaker baseline performance on measures of speech production would be associated with greater improvement tDCS. Finally, integrating the above two hypotheses, we predicted that the relationship between baseline severity and response to tDCS would be most robust in measures of word level production, the aspect of spontaneous speech that we expected to be most responsive to therapeutic neuromodulation.

## Methods

### Overview

This was a two-phase study. In the first phase the optimal stimulation montage was identified. The second phase introduced tDCS as a treatment, utilizing a sham-controlled partial crossover design with 2 weeks (10 days) of stimulation followed by a 2-week and a 2-month follow-up. The methods summarized here are described in more detail in our previous work (Shah-Basak et al., 2015).

### Subjects

**Figure 1:**
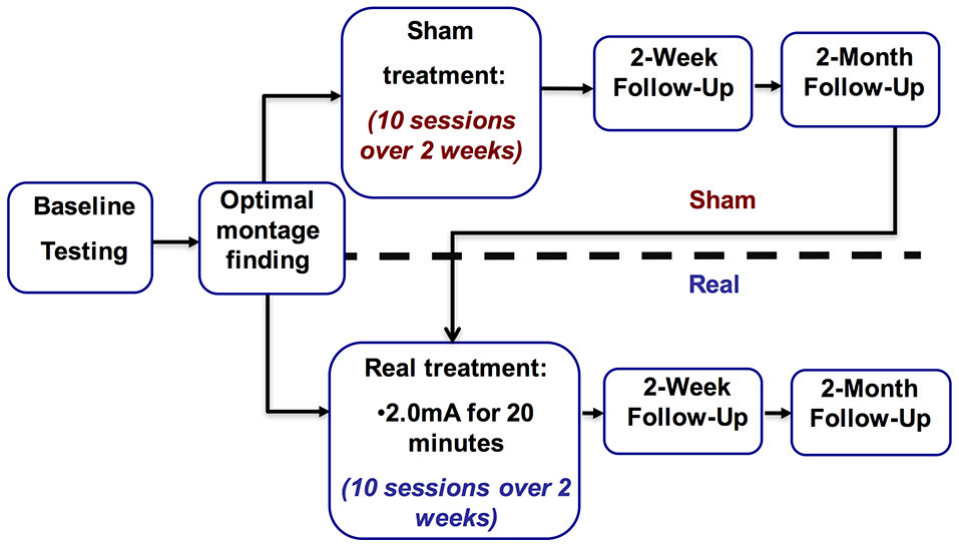
Study overview.

Subjects had a history of a first time single left-hemispheric chronic stroke (≥6 months post-stroke-onset), had mild-to-severe non-fluent aphasia, were premorbidly right-handed (Edinburgh Handedness Inventory) (Oldfield, 1971), and had no concurrent history of neurological, psychiatric or unstable medical conditions, or any contraindication to either MRI or tDCS (Table 1). Aphasia symptoms and severity were screened using the Western Aphasia Battery (WAB) (Kertesz, 1982), to avoid ceiling effects, individuals with a WAB-Aphasia Quotient (WAB-AQ) above 90 were excluded. Out of 26 screened subjects, 3 were medically ineligible, 5 did not meet the eligibility criterion, and 1 was lost to follow-up, resulting in 11 enrolled subjects, and 9 of which progressed to phase 2 (2 females; age: 62.0 ± 10.8, range = 53–84 years; Figure 1). None of the enrolled subjects initiated new language therapies or engaged in other treatment studies during the course of the study. A single neurologist (RHH) used clinical scans (MRI/CT) obtained during or after each patient's medical treatment for stroke to delineate lesion locations. The study was approved by the Institutional Review Board of the University of Pennsylvania, and each subject, or his or her legally authorized representative, provided informed consent.

**Table 1:** Subject demographics.

**Subject**	**Sex**	**Age, y**	**Time since stroke, mo**	**Type of stroke**	**Lesion distribution**	**Lesion volume (cm^3^)**	**WAB-AQ**	**Optimal montage**	**Number of nouns (baseline)**	**Number of nouns (2 weeks)**	**Number of nouns (2 months)**
**REAL tDCS**											
R1	M	65	27	Ischemic	Left MCA	~	29.1	Anode F3	0		0
S1/R2	M	53	67	Ischemic	Fronto-parietal cortical and subcortical, including internal capsule, basal ganglia, anterior IFG	165.49	87.8	Anode F3	14	20	14
R3	M	54	8	Ischemic	Large fronto-temporo-parietal lesion involving STG, parietal cortex, IFG, and subcortical white matter Caudate and thalamus spared	271.02	38.9	Cathode F3	4	8	1
R4	M	76	100	Ischemic	Fronto-temporo-parietal subcortical, including corona radiata Internal capsule, deep gray structures, and IFG spared	145.94	69.6	Cathode F3	27	27	17
S2/R5	M	61	28	Hemorrhagic	Fronto-parietal lesion involving sensorimotor and superior parietal cortices, and subcortical white matter IFG, inferior parietal gyrus, temporal cortex, deep gray structures, and thalamus spared	134.04	83.0	Cathode F4	30	20	23
S4/R6	M	67	10	Ischemic	Fronto-parietal lesion involving supramarginal gyrus, temporo-parietal-occipital junction, insula, IFG, and underlying subcortical white matter Basal ganglia and thalamus spared	89.8	69.5	Cathode F3	22	19	19
S5/R7	F	84	26	Ischemic	Left MCA	~	78.1	Anode F3	8	8	9
S6/R8	F	50	116	Ischemic	Left MCA	~	78.7	Anode F4	14	15	15
Mean (StdDev)		62.0 (±1 0.8)	50.5 (± 41.2)						15 (± 10.7)	17 (± 6.9)	12 (± 8.3)
**SHAM tDCS**											
S1/R2	M	53	67	Ischemic					14	12	15
S2/R5	M	61	28	Hemorrhagic					30	22	23
S3	M	61	12	Ischemic	Large fronto-temporo-parietal lesion involving STG, parietal cortex, left IFG and subcortical white matter Deep gray structures and thalamus spared	266.29	23.2		1	0	0
S4/R6	M	67	10	Ischemic					22	19	15
S5/R7	F	84	26	Ischemic					8	5	10
S6/R8	F	50	116	Ischemic					14	12	17
Mean (StdDev)		57.3 (± 4.6)	35.7 (±2 8.3)						15 (± 10.1)	12 (± 8.3)	13 (± 7.8)

*MCA, Middle cerebral artery; IFG, Inferior frontal gyrus; STG, Superior temporal gyrus; SD, Standard deviation. Of note, structural images were reviewed during subject screening and enrolment but were not available during data analysis or results reporting (~). Additionally, areas in gray under the sham tDCS section represent previously stated demographics for the same subjects in the real tDCS*.

### Transcranial direct current stimulation

As described in our earlier paper (cf. Shah-Basak et al., 2015; Figure 1), the stimulation paradigm is as follows. In both phases of the study we used a Magstim Eldith 1 Channel DC Stimulator Plus (Magstim, Whitland, UK). A recent review by Bikson and colleagues explored the safety of tDCS. They defined conventional tDCS protocols as ≤40 min, ≤4 miliamperes, and ≤7.2 Coulombs. This review covered 33,200 sessions and 1,000 subjects and found no reports of serious adverse effect or irreversible injury after repeated sessions (Bikson et al., 2016). In line with widely used and safe parameters (Brunoni et al., 2011; Kessler et al., 2012; Russo et al., 2013; Bikson et al., 2016), stimulation was delivered for 20 min at 2.0 mA using 5 × 5 cm^2^ sponge electrodes (current density: 0.80 μA/mm^2^) with a 30-s ramp-up and ramp-down period. For sham, stimulation was ramped up to 2.0 mA and then down to 0 mA in the first minute of stimulation, and subjects were randomized to either receive tDCS with either the anode or cathode over either the left frontal lobe or right frontal lobe, or sham stimulation, leading to a total five conditions (i.e., anode left, cathode left, anode right, cathode right, and sham). In all conditions, the countervailing electrode was positioned over the contralateral mastoid. The order of five conditions was counterbalanced across subjects, who were blinded to whether they were receiving real or sham-tDCS (Gandinga et al., 2006). The person administering tDCS was not blinded to tDCS conditions.

### Phase 1: optimal montage identification

Over five non-consecutive days, subjects underwent tDCS with the four active conditions and one sham condition, one condition per session. These sessions were separated on average by at least 5 days. Frontal lobe stimulation sites were identified using the 10–20 EEG measurement system (F3 = left; F4 = right). Thus, the active conditions were F3-anode, F3-cathode, F4-anode, and F4-cathode. These frontal sites overlie brain areas that are superior to the inferior frontal gyrus, which is often lesioned in patients with non-fluent aphasia. We theorized that F3 stimulation would likely be associated with perilesional stimulation in the left hemisphere.

Previously described in our earlier paper (cf. Shah-Basak et al., 2015), picture-naming ability was assessed before and immediately after each stimulation session with an 80-item task using images from the International Picture Naming Project database (IPNP) (Szekely et al., 2004). The 80-item picture sets were matched for word-frequency, word-length, and semantic category. Different item lists were assigned to each days and to the pre- and post- tDCS assessment. The difference between the number of items that were named correctly before and following each stimulation session was calculated (post- vs. pre-stimulation). To examine variability in responsiveness to tDCS, we first compared the change in subjects' performance across all active montages with respect to the sham montage. Second, in line with previously reported methods (Naeser et al., 2005; Medina et al., 2012), an electrode montage was defined as optimal for each subject if the subject (1) showed the greatest change in accuracy after stimulation using a particular montage and (2) if the accuracy post-stimulation with that montage was ≥ the upper limit of the 90% confidence interval (CI) of pre-stimulation performance across all montages.

### Phase 2: stimulation of individually-selected montages

At the conclusion of Phase 1, 11/26 subjects exhibited significant transient improvement in naming after stimulation with at least one active electrode arrangement. Ten subjects entered the sham controlled partial crossover portion of the study; one subject declined further study participation. Another subject completed only the sham arm, but declined to participate in the real-tDCS phase. The data included in this analysis is from the 9 subjects that participated in phase 2. Each of the 9 subjects was randomized to receive either real-tDCS treatment only (*N* = 3), or sham stimulation followed by real-tDCS (*N* = 6). There were no significant differences in the demographics of the 9 subjects apart from their initial severity at baseline (2 females; age: 62.0 ± 10.8, range = 53–84 years; baseline WAB: 62.0, range 23.2–87.8).

**Figure 2:**
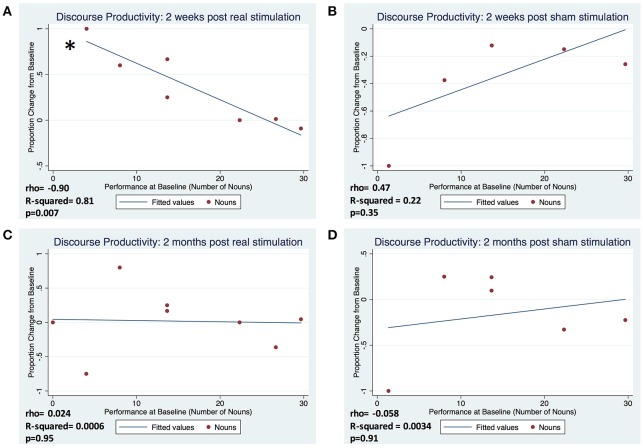
Proportion change in baseline of discourse productivity according to average baseline performance, represented by nouns, at 2 weeks following real **(A)** and sham **(B)** stimulation and 2 months following real **(C)** and sham **(D)** stimulation. Proportion change from baseline was calculated as: (follow-up performance–baseline performance)/baseline performance. (^*^Represents *p* < 0.05).

To establish a stable pre-tDCS baseline of aphasia severity, the Cookie Theft narrative picture description was administered 3 times in separate behavioral sessions prior to initiating real or sham treatment. During treatment, subjects received tDCS for a total of 10 days (Monday–Friday for two consecutive weeks). Stimulation parameters were identical to those described during optimal montage identification. Subjects engaged in the training task described above during both the real- and sham-tDCS sessions (Maher et al., 2006). Subjects repeated the assessment with the Cookie Theft narrative picture description at 2 weeks and 2 months after treatment. Following 2-month follow-up, subjects in the sham arm crossed over into the real arm and received real-tDCS, followed by 2-week and 2-month follow-up assessments (Figure 2). Subjects who initially received real-tDCS were blinded to their treatment condition. Subjects receiving sham stimulation were blinded to their condition until they crossed over into the real arm of the study, at which point they were by necessity informed of their condition (as required by our IRB).

#### Language training task

As described in our earlier paper (cf. Shah-Basak et al., 2015), during the 20 min of active- or sham-tDCS, subjects completed a picture-naming task that was based on (but was not identical to) constraint-induced language therapy (CILT), in that it minimized non-verbal communication between subjects and the experimenter (Pulvermuller et al., 2001; Maher et al., 2006). Subjects were shown 20 black-and-white images taken from the IPNP database, one at a time. A physical barrier between subjects and the experimenter was erected to constrain subjects to produce verbal responses and also to prevent unanticipated visual cues from the experimenter (Maher et al., 2006).

#### Measures of fluency

The Cookie Theft narrative picture description is a subtest of the Boston Diagnostic Aphasia Examination (BDAE, Goodglass et al., 2001) that measures spontaneous speech—a combination of information content and fluency. At baseline and at each of the follow-up time points, subjects described the Cookie Theft picture stimulus. They were not given a time limit. Their responses were digitally recorded, then stripped of all identifiers, and transcribed.

The spontaneous speech or fluency component of the Cookie Theft can be analyzed with respect to 3 distinct conceptual areas: (1) production, elaboration, and complexity, (2) conciseness, and (3) information imparted. These areas were assessed using Quantitative Production Analysis (QPA; Saffran et al., 1989). We categorized these variables based on four aspects of speech fluency: discourse (i.e., word level) productivity, sentence productivity, grammatical accuracy, and lexical selection (Gordon, 2006). In this study, these four aspects of speech were represented in our analysis by four specific measures: the number of nouns generated, sentence length, the proportion of well-formed sentences, and the proportion of pronouns, respectively.

#### Analysis

STATA was used for all statistical analyses. In order to determine if subjects that received real stimulation improved significantly from baseline compared to those that received sham, we conducted a group analysis using Wilcoxon ranked sign test. In this analysis we measured changes in performance between baseline and 2-week and 2-month follow-up, contrasting subjects who received real and sham tDCS. We used a non-parametric test because our sample size was small, and as a result we were unable to determine whether the data could be distributed normally. In this analysis all those that received real stimulation composed one group and all those that received sham composed a separate group. The threshold of significance for this initial analysis was a *p* ≤ 0.05.

We subsequently used Spearman correlations to explore associations between the degree of language improvement on each of our measures and baseline aphasia severity. The degree of language improvement was measured by change from baseline at the 2-week and the 2-month follow-up sessions for the 9 subjects - those that received real stimulation, less the one subject that that withdrew after only completing sham (*N* = 8) and those that received sham (*N* = 6) stimulation.

Finally, because this was a partial crossover study, one potential concern was that subjects who had received only real stimulation might have systematically performed differently than those who had received sham followed by real stimulation. In order to evaluate this possibility, we conducted Mann-Whitney U tests to compare performance in these two subgroups. Once again, the threshold of significance was a *p* ≤ 0.05.

## Results

### tDCS and measures of fluency

Using a Wilcoxon ranked sign test to be assessed the within group effect of tDCS on measures of fluency. We found no significant change from baseline performance at the 2-week follow up compared to no change [discourse productivity (*p* = 0.35), sentence length (*p* = 0.08), proportion of well-formed sentences (*p* = 0.40), and proportion of pronouns (*p* = 0.74)] or at the 2 month follow up [discourse productivity (*p* = 0.40), sentence length (*p* = 0.09), proportion of well-formed sentences (*p* = 0.12), and proportion of pronouns (*p* = 0.67)].

### Influence of baseline severity on tDCS effects

In subjects that completed one or both arms of the study (real and sham), factors of interest were separated by QPA categories—discourse productivity, sentence productivity, grammatical accuracy, and lexical selection. We demonstrated a very strong correlation between number of nouns produced at baseline and the change from baseline at 2 weeks post-real stimulation (ρ = −0.90, R-squared = 0.81, *p* = 0.007) compared to the 2 weeks post-sham stimulation (ρ = 0.47, R-squared = 0.22, *p* = 0.35). However, this pattern of association was not maintained at 2 months post-real stimulation (ρ = 0.024, R-squared = 0.0006, *p* = 0.95) and sham (ρ = −0.058, R-squared = 0.0034, *p* = 0.91) (Figure 2). There were no significant correlations between severity of baseline performance and degree of improvement at 2 weeks or 2 months in any of the other measures of fluency (sentence lengths, proportion of well-formed sentences, and proportion pronouns; all *p*'s > 0.5). Similarly, no significant correlations were observed for sham stimulation for any of the outcome measures at 2 weeks or 2 months post-stimulation (all *p*'s > 0.5) (Table 2). Additionally, we did not appreciate a significant correlation between years of education and degree of improvement from baseline at the 2 week or 2 month follow-ups (ρ = −0.691, *p* = 0.13) [2 weeks] (ρ = −0.572, *p* = 0.18) [2 months]. Pearson correlations were used to explore associations between the degree of language fluency improvement and education.

**Table 2:** Proportion change in baseline of sentence productivity, grammatical accuracy, and lexical selection according to average baseline performance, represented by mean sentence length, proportion of well-formed sentences, and proportion proportion of pronouns at 2 weeks and 2 months following real and sham stimulation.

	**Simulation type**	**Time point**	**Spearman correlation**		
			**ρ**	***R* squared**	***p*-value**
Discourse Productivity: Number of nouns	Real	2 weeks	−0.9009	0.8116	0.0056
		2 months	0.0241	0.0006	0.9548
	Sham	2 weeks	0.4706	0.2215	0.3462
		2 months	−0.058	0.0034	0.9131
Sentence Productivity: Mean sentence length	Real	2 weeks	−0.0748	0.0056	0.8734
		2 months	0.4419	0.1953	0.273
	Sham	2 weeks	−0.1715	0.0294	0.7453
		2 months	−0.1715	0.0294	0.7453
Grammatical Accuracy: Proportion well-formed sentences	Real	2 weeks	0.5714	0.3265	0.1802
		2 months	0.4791	0.2295	0.2297
	Sham	2 weeks	−0.4058	0.1647	0.4247
		2 months	−0.6	0.3600	0.208
Lexical Selection: Proportion of pronouns	Real	2 weeks	0.2143	0.0459	0.6103
		2 months	0.2143	0.0459	0.6103
	Sham	2 weeks	−0.2571	0.0661	0.6228
		2 months	−0.2571	0.0661	0.6228

Proportion change from baseline was calculated as: (follow-up performance – baseline performance)/baseline performance. ^*^Represents p < 0.05.

Observing a robust correlation between baseline severity and change on discourse productivity resulting from tDCS, we further quantified the influence of low and high baseline language ability by comparing to mean change in noun production in the 4 patients with the most severe baseline aphasia to that of the 4 least severe patients in both real and sham conditions. Because the exploratory analysis was in small samples and was driven by the observation of a strong directional relationship, employed a one-tailed Mann-Whitney U test. Patients with more severe baseline aphasia responded positively (*p* = 0.038) to tDCS in measures of word-level production at 2 weeks when compared to the less severe group at 2 weeks (*p* = 0.14). This difference was not found in the sham condition for either more or less severe patients.

We used a Mann-Whitney U analysis to demonstrate that there were no significant differences in performance between subjects who received sham stimulation followed by real tDCS (*n* = 6) and those that only received real stimulation (*n* = 3) on any of the study measures at either the 2-week or 2-month follow up time points (all *p*'s > 0.5). Finally, of the 8 subjects who received real stimulation, we note that there was a fairly even distribution between those that received sham stimulation followed by real and those that only received real tDCS with respect to baseline severity. In the most severe group 2 of the 4 received sham prior to real and in the least severe group 3 of the 4 subjects received sham prior to real stimulation.

The initial lesion volume assessment was completed with lesion tracings of pre-stimulation MRI images from the post-stroke population. Lesion volumes were calculated in cm^3^ for the 7 of the 9 subjects. 2 subjects' initial MRI had excessive motion artifact, which made an accurate calculation of the volume impossible. For those that received real tDCS (*n* = 7), there was a mean lesion volume of 161.26 cm^3^ (±67.36 cm^3^). And for those that received sham tDCS (*n* = 5), there was a mean lesion volume of 163.91 cm^3^ (±74.99 cm^3^). Pearson correlations were used to explore associations between the degree of language fluency improvement and lesion volume. The degree of language improvement was measured by change from baseline at the 2-week follow-up. We demonstrated a very strong correlation between lesion volume and change in number of nouns produced at 2 weeks post-real stimulation (ρ = 0.81, *p* = 0.05) compared to the 2 weeks post-sham stimulation (ρ = 0.69, *p* = 0.31).

Regarding the time since stroke, for those that received real tDCS (*n* = 8), there was a mean time post stroke of 48 months (±41.9 months). And for those that received sham tDCS (*n* = 6), there was a mean lesion volume of 43 months (±41.0 months). Pearson correlations were used to explore associations between the degree of language fluency improvement and time post stroke. The degree of language improvement was measured by change from baseline at the 2-week follow-up. There was no correlation between time post stroke and change in number of nouns produced at 2 weeks or 2 months post-real stimulation (ρ = −0.266, *p* = 0.56) [2 weeks] (ρ = 0.096, *p* = 0.82) [2 months] compared to the 2 weeks or 2 months post-sham stimulation (ρ = 0.50, *p* = 0.32) [2 weeks] (ρ = 0.62, *p* = 0.19) [2 months].

## Discussion

The current study focused on identifying factors that can predict recovery of language fluency and which aspects of language fluency can be expected to improve with the identified factor(s). Influenced by previous studies in different patient populations that sought to establish factors that influence response to tDCS (Turkeltaub et al., 2012; Furuya et al., 2014; Uehara et al., 2015), we predicted that baseline severity would influence the improvement in chronic non-fluent aphasic patients. Our study demonstrated that there was an association between language severity at baseline and degree of improvement post-stimulation. We also predicted that there would be improvement at the word level, because of our previous rTMS study demonstrated that after therapeutic neuromodulation, language improvement was seen only in measures of discourse (word level) productivity (Medina et al., 2012). The pattern of language improvement we observed, in which only those who were more severely affected at baseline improved, was seen only in the category of discourse productivity. In other words, participants improved only on measures of word level production, and only if their initial presentation at baseline was severe. This association was striking, accounting for 81% of the variability of performance of the cohort. Additionally, because the groups were fairly evenly distributed between subjects who only received real and those that received sham followed by real and our analysis did not reflect a significant difference between them, it is unlikely that the correlation we identified can be attributed to an order effect. Of note, group analyses, which compared all those that who received real stimulation to those who received sham stimulation, were negative. The fact that the pattern of improvement was only observed when the groups were separated by severity, underscores the importance of elucidating the factors that influence response to tDCS.

We found that, while there was a wide range in severity as measured by the WAB aphasia quotient (WAB-AQ), there was no correlation between initial WAB performance and fluency outcomes, nor was there a clear correlation between age and fluency outcomes. It is possible then that the correlation that was seen between baseline severity in discourse productivity and degree of improvement in that measure was task specific. In other words, one's severity in a discourse productivity task may predict one's degree of improvement in that specific aspect of language. The WAB-AQ, however, contains measures of discourse productivity in addition to other categories of language assessment. It is therefore possible that the severity of one's WAB-AQ may not be predictive of degree of improvement in discourse productivity as an isolated measure.

While a few prior investigations have evaluated predictors of language recovery after stroke, these have largely focused on the acute stage of the disease (Lazar et al., 2008, 2010), since it is generally acknowledged that the majority of language improvement occurs approximately in the first 3 months after stroke (Robey, 1998; Berthier, 2005). Contrary to what we found in our study, several studies have suggested that baseline aphasia severity is a negative predictor of language recovery (Laska et al., 2001; Pedersen et al., 2004; Lazar et al., 2010). Many of these studies, however, focused on patients in the acute setting (Fillingham et al., 2006; Lazar et al., 2010), did not involve any additional intervention, and were not specific to aphasia type (Pedersen et al., 2004). Our finding however, has potential implications for interventions in chronic and more severely affected individuals.

To our knowledge no prior studies have evaluated the impact of baseline severity on response to neuromodulation therapy in the chronic non-fluent aphasic population. Previously the relationship between baseline severity and response to tDCS has been reported primarily in healthy subjects, but not in patient populations (Turkeltaub et al., 2012; Furuya et al., 2014; Sarkar et al., 2014; Uehara et al., 2015). For this chronic patient population, the possibility that more severe initial language deficits are associated with greater improvement introduces the possibility that more severe patients may have a recovery window that extends beyond a traditional 3-month recovery period. Importantly, our results demonstrate that specific symptoms, like word level production deficits, that respond to treatment can be identified. Furthermore, we found that patient subgroups respond differently to tDCS, wherein the worse affected patients improve more in word-level production compared to milder patients. Overall this could be important for appropriate stratification in clinical studies and may someday influence clinical care.

Although ultimately in line with our predictions, it is intriguing that the association between baseline severity and post-tDCS change was very high for word-level production and non-existent for other fluency measures. One possible way to account for this stark disparity is to consider the role that the cognitive task performed during stimulation might have had on post-stimulation behavioral changes. We have previously observed in healthy subjects that the degree to which a cognitive training task engages particular mental abilities during tDCS directly influences the extent to which performance on tasks that require similar abilities are affected by stimulation (Gill et al., 2015). In the current study, patients received stimulation while they were performing a picture-naming task using a protocol that constrained them to communicate by producing verbal responses (Maher et al., 2006). The pictures being named were all objects (i.e., nouns). One possibility is that the nature of the training performed during tDCS specifically reinforced language production at the word level, and perhaps even more specifically the generation of nouns. While this notion of near transfer between related tasks may be an attractive account, strong confirmation of this hypothesis would require further experiments involving manipulation of the training task in order determine whether other aspects of fluency could be selectively influenced.

Considering the results of our prior work in this cohort of subjects demonstrated an improvement in overall aphasia severity that was maintained at 2 months (Shah-Basak et al., 2015), we did not expect our improvement to be limited to 2 weeks after stimulation. The primary difference between these two studies is how language improvement was measured. In the previous study the WAB aphasia quotient was used. This is a composite measure of several language domains including fluency as well as comprehension, repetition, and naming. In the current study, however, we evaluated only aspects of language fluency as measured by changes in spontaneous elicited speech. It is possible then that the improvement that was maintained at 2 months in our previous study was mediated by multiple domains of language production and not fluency alone.

There are clear limitations in this study. Most notably, the study employed a small sample size. Several factors contributed to this. First, given the relatively high rate of exclusion from the study, enrolling a large number of subjects with chronic aphasia proved challenging. Elements of the study design also limited the number of subjects who participated. For instance, the only subjects who participated in phase 2 of the study were those that had an optimal montage identified in phase 1, further limiting the sample size. Participation in the study also required a considerable time commitment; it took over 2 months to complete the real arm and at least an additional 2 months to complete the sham followed by the real arm. This resulted in one subject withdrawal. Additionally, this study was designed as a partial crossover. This designed allowed all subjects to receive real-tDCS eventually, however, it also resulted in unequal subject groups. This complicated the direct comparison between the real and sham conditions. It was helpful to demonstrate that the real only and sham-then-real data were similar to one another and thus collapsible, however, future studies should to follow a full crossover design then compare the two groups. Importantly, future studies should also employ sample sizes that are sufficient to provide greater statistical power. However, we would also note that we have, in previously published work, been able to demonstrate a significant effect of non-invasive brain stimulation on language ability in cohorts of persons with aphasia with similarly small sample sizes - 6 and 10 (Medina et al., 2012; Shah-Basak et al., 2016). In these studies, we were able to demonstrate an effect on overall aphasia severity following tDCS (Shah-Basak et al., 2016) and an effect on fluency following TMS (Medina et al., 2012).

In recent literature there has been some discussion regarding the potential lasting effects of single session tDCS. Single session tDCS has been shown to have an immediate transient effect in various cognition related tasks (Kekic et al., 2017). The effects of tDCS have been observed up to an hour following a single stimulation session and with repeated stimulation may persist for days or even months after multiple days of stimulation (Reis et al., 2009). A recent study suggests that there may be a delayed cognitive effect on multitasking tasks after receiving a single course of tDCS (Nelson et al., 2016). In our study, however, when establishing optimal stimulation parameters all subjects returned to within 2 standard deviations of their pre-stimulation baseline scores prior to proceeding with the next montage suggesting that the improved that they experienced after a single course of stimulation was only transient.

There have been several recent reviews that have explored the distant effects of non-invasive brain stimulation (Siebner et al., 2009; Siebner and Ziemann, 2010). More recently a study by Polania and colleagues used a graph theoretical approach to evaluate the effects of tDCS on fMRI connectivity. This analysis demonstrated that anodal tDCS over M1 reduced the functional connectivity between the stimulated M1 and the premotor and superior parietal regions (Polanía et al., 2011). The same group later showed that anodal tDCS of the M1 also increased connectivity between the stimulated region and the ipsilateral subcortical regions (Polania et al., 2012). These findings have been supported by MRI profusion studies (Stagg et al., 2013). Literature supports an effect of montage regarding the distant effects of tDCS on cortical connectivity. Sehm et al. demonstrated during bilateral, and non-unilateral, tDCS resting state changes can be seen in both local and distant areas (Sehm et al., 2013). Our study employed unilateral tDCS in presumed reorganized language regions. While the effects of tDCS in this context may be mediated by remote connections, this issue as it related to this patient population and stimulation approach has yet to be fully explored.

Currently there is controversy regarding the reliability of the effect of tDCS. Some have argued that the effects of tDCS on cognition and neurophysiology are modest, highly variable, or possibly even non-existent (e.g., Horvath et al., 2015, 2016, but also Price et al., 2015). One of the primary challenges in making inferences about the efficacy of tDCS is that investigators have yet to define which specific aspects of behavioral performance are most likely to be influenced by tDCS. Additionally, it is not yet clear which subject characteristics may predispose them to respond differentially to tDCS. Elucidating these fundamental properties will prove especially important as tDCS is employed increasingly in clinical studies and perhaps someday in clinical care. Future studies, especially clinical investigations, will need to replicate and extend analyses like these in order to better address who will benefit from stimulation and which specific deficits can be influenced.

## Ethics statement

University of Pennsylvania Institutional Review Board. Informed consent was obtained by the PI or other designated members of the research team. Because this protocol involved the enrollment of subjects who are known to have language deficits, some subjects had difficulty understanding what has been explained to them about the protocol, either verbally or in writing. In other cases, subjects with relatively mild deficits or deficits restricted to the domain of language production were able to understand what has been explained to them quite readily. In cases where individuals suffer from deficits of verbal or reading comprehension (as assessed by the PI, a behavioral neurologist) we required that informed consent be obtained from both the patient and a legally authorized representative. The Informed Consent (IC) form was provided to the subject (and to their legally authorized representative, when needed) and was reviewed in detail by the PI or another designated member of the research team. After reading and verbally reviewing the document, the subject (and their representative, as needed) were asked if there are any questions or concerns. If the subject (and their representative, as needed) indicated agreement with the participation by signing the ICF, indicated that there were no additional questions, and met inclusion/exclusion criteria, the subject was included in the study. In cases where subjects have intact language comprehension and do not require a legally authorized representative, we documented on the consent form that a cosignatory by such an individual is not needed by writing or N/A on the signature line of the legally authorized representative. All subjects were told in clear and explicit terms that they are not required to participate in the study. They were also explicitly told that not participating in the study or withdrawing from the study at any time would have no adverse consequences in any respect to their future care or standing with the University of Pennsylvania System or Medical School. Subjects were told that if they wish to withdraw from the study at any time, including during the tDCS application, they are free to do so; the study would be terminated immediately. They were also told that they would be paid for their time should they withdraw. All of this information was also made clear to the subjects' legally authorized representative.

## Author contributions

CN contributed substantially to the conception of the work, was responsible for data collection, drafting the manuscript, and agrees to be accountable for all aspects of the work in ensuring that questions related to the accuracy or integrity of any part of the work are appropriately investigated and resolved. DS contributed substantially to the conception of the work, was responsible for data collection, and agrees to be accountable for all aspects of the work in ensuring that questions related to the accuracy or integrity of any part of the work are appropriately investigated and resolved. RH contributed substantially to the conception of the work, was responsible for revising the manuscript critically for important intellectual content, and agrees to be accountable for all aspects of the work in ensuring that questions related to the accuracy or integrity of any part of the work are appropriately investigated and resolved.

### Conflict of interest statement

The authors declare that the research was conducted in the absence of any commercial or financial relationships that could be construed as a potential conflict of interest.

## References

[B1] Baker J. M. Rorden C. Fridriksson J. (2010). Using transcranial direct-current stimulation to treat stroke patients with aphasia. Stroke 41, 1229–1236. 10.1161/STROKEAHA.109.576785 20395612 PMC2876210

[B2] Berthier M. L. (2005). Poststroke aphasia: epidemiology, pathophysiology and treatment. Drugs Aging 22, 163–182. 10.2165/00002512-200522020-00006 15733022

[B3] Bikson M. Grossman P. Thomas C. Zannou A. L. Jiang J. Adnan T.. (2016). Safety of transcranial direct current stimulation: evidence based update 2016. Brain Stimul. 9, 641–661. 10.1016/j.brs.2016.06.004 27372845 PMC5007190

[B4] Boggio P. S. Nunes A. Rigonatti S. P. Nitsche M. A. Pascual-Leone A. Fregni F. (2007). Repeated sessions of noninvasive brain DC stimulation is associated with motor function improvement in stroke patients. Restor. Neurol. Neurosci. 25, 123–129. 17726271

[B5] Brunoni A. R. Ferrucci R. Bortolomasi M. Vergari M. Tadini L. Boggio P. S.. (2011). Transcranial direct current stimulation (tDCS) in unipolar vs. bipolar depressive disorder. Prog. Neuropsychopharmacol. Biol. Psychiatry 35, 96–101. 10.1016/j.pnpbp.2010.09.010 20854868

[B6] Brunoni A. R. Nitsche M. A. Bolognini N. Bikson M. Wagner T. Merabet L.. (2012). Clinical research with transcranial direct current stimulation (tDCS): challenges and future directions. Brain Stimul. 5, 175–195. 10.1016/j.brs.2011.03.002 22037126 PMC3270156

[B7] Cappon D. Jahanshahi M. Bisiacchi P. (2016). Value and efficacy of transcranial direct current stimulation in the cognitive rehabilitation: a critical review Since 2000. Front Neurosci. 10:157. 10.3389/fnins.2016.00157 27147949 PMC4834357

[B8] Fillingham J. K. Sage K. Lampoon Ralph M. A. (2006). The treatment of anomia using errorless learning. Neuropsychol. Rehabil. 16, 129–154. 10.1080/09602010443000254 16565031

[B9] Fiori V. Coccia M. Marinelli C. V. Vecchi V. Bonifazi S. Ceravolo M. G.. (2011). Transcranial direct current stimulation improves word retrieval in healthy and nonfluent aphasic subjects. J. Cogn. Neurosci. 23, 2309–2323. 10.1162/jocn.2010.21579 20946060

[B10] Flöel A. Meinzer M. Kirstein R. Nijhof S. Deppe M. Knecht S.. (2011). Short-term anomia training and electrical brain stimulation. Stroke 42, 2065–2067. 10.1161/STROKEAHA.110.609032 21636820

[B11] Fridriksson J. Richardson J. D. Baker J. M. Rorden C. (2011). Transcranial direct current stimulation improves naming reaction time in fluent aphasia: a double-blind, sham-controlled study. Stroke 42, 819–821. 10.1161/STROKEAHA.110.600288 21233468 PMC8210639

[B12] Furuya S. Klaus M. Nitsche M. A. Paulus W. Altenmuller E. (2014). Ceiling effects prevent further improvement of transcranial stimulation in skilled musicians. J. Neurosci. 34, 13834–13839. 10.1523/JNEUROSCI.1170-14.2014 25297109 PMC6608385

[B13] Goodglass H. Kaplan E. Barresi B. (2001). Boston Diagnostic Aphasia Examination, 3rd Edn. Philadelphia, PA: Lippincott Williams & Wilkins.

[B14] Gandinga P. C. Hummel F. C. Cohen L. G. (2006). Transcranial DC stimulation (tDCS): a tool for double-blind sham-controlled clinical studies in brain stimulation. Clin. Neurophysiol. 117, 845–850. 10.1016/j.clinph.2005.12.003 16427357

[B15] Gill J. Shah-Basak P. P. Hamilton R. (2015). It's the thought that counts: examining the task-dependent effects of transcranial direct current stimulation on executive function. Brain Stimul. 8, 253–259. 10.1016/j.brs.2014.10.018 25465291

[B16] Gordon J. (2006). A quantitative production analysis of picture description. Aphasiology 20, 188–204. 10.1080/02687030500472777

[B17] Heiss W. D. Thiel A. (2006). A proposed regional hierarchy in recovery of post-stroke aphasia. Brain Lang. 98, 118–123. 10.1016/j.bandl.2006.02.002 16564566

[B18] Horvath J. C. Forte J. D. Carter O. (2015). Quantitative review finds no evidence of cognitive effects in healthy populations from single-session transcranial direct current stimulation (tDCS). Brain Stimul. 8, 535–550. 10.1016/j.brs.2015.01.400 25701175

[B19] Horvath J. C. Vogrin S. J. Carter O. Cook M. J. Forte J. D. (2016). Effects of a common transcranial direct current stimulation (tDCS) protocol on motor evoked potentials found to be highly variable within individuals over 9 testing sessions. Exp. Brain Res. 234, 2629–2642. 10.1007/s00221-016-4667-8 27150317

[B20] Kekic M. McClelland J. Bartholdy S. Boysen E. Musiat P. Dalton B.. (2017). Single-session transcranial direct current stimulation temporarily improves symptoms, mood, and self-regulatory control in bulimia nervosa: a randomised controlled trial. PLoS ONE 12:e0167606. 10.1371/journal.pone.0167606 28121991 PMC5266208

[B21] Kertesz A. (1982). The Western Aphasia Battery: Test Manual, Stimulus Cards, and Test Booklets. New York, NY: Grune & Stratton.

[B22] Kessler S. K. Turkeltaub P. E. Benson J. G. Hamilton R. H. (2012). Differences in the experience of active and sham transcranial direct current stimulation. Brain Stimul. 5, 155–162. 10.1016/j.brs.2011.02.007 22037128 PMC3270148

[B23] Kyrozis A. Potagas C. Ghika A. Tsimpouris P. K. Virvidaki E. S. Vemmos K. N. (2009). Incidence and predictors of post-stroke aphasia: the Arcadia Stroke Registry. Eur. J. Neurol. 16, 733–739. 10.1111/j.1468-1331.2009.02580.x 19475755

[B24] Laska A. C. Hellblom A. Murray V. Kahan T. Von Arbin M. (2001). Aphasia in acute stroke and relation to outcome. J. Intern. Med. 249, 413–422. 10.1046/j.1365-2796.2001.00812.x 11350565

[B25] Lazar R. M. Minzer B. Antoniello D. Festa J. R. Krakauer J. W. Marshall R. S. (2010). Improvement in aphasia scores after stroke is well predicted by initial severity. Stroke 41, 1485–1488. 10.1161/STROKEAHA.109.577338 20538700 PMC2921806

[B26] Lazar R. M. Speizer A. E. Festa J. R. Krakauer J. W. Marshall R. S. (2008). Variability in language recovery after first-time stroke. J. Neurol. Neurosurg. Psychiatr. 79, 530–534. 10.1136/jnnp.2007.122457 17846113

[B27] Maher L. M. Kendall D. Swearengin J. A. Rodriguez A. Leon S. A. Pingel K.. (2006). A pilot study of use-dependent learning in the context of Constraint Induced Language Therapy. J. Int. Neuropsychol. Soc. 12, 843–852. 10.1017/S1355617706061029 17064447

[B28] Medina J. Norise C. Faseyitan O. Coslett H. B. Turkeltaub P. E. Hamilton R. H. (2012). Finding the right words: transcranial magnetic stimulation improves discourse productivity in non-fluent aphasia after stroke. Aphasiology 26, 1153–1168. 10.1080/02687038.2012.710316 23280015 PMC3532848

[B29] Mimura M. Kato M. Sano Y. Kojima T. Naeser M. Kashima H. (1998). Prospective and retrospective studies of recovery in aphasia changes in cerebral blood flow and language functions. Brain 121, 2083–2094. 10.1093/brain/121.11.2083 9827768

[B30] Monti A. Cogiamanian F. Marceglia S. Ferrucci R. Mameli F. Mrakic-Sposta S.. (2008). Improved naming after transcranial direct current stimulation in aphasia. J. Neurol. Neurosurg. Psychiatr. 79, 451–453. 10.1136/jnnp.2007.135277 18096677

[B31] Naeser M. A. Martin P. I. Nicholas M. Baker E. H. Seekins H. Helm-Estabrooks N.. (2005). Improved naming after TMS treatments in a chronic, global aphasia patient–case report. Neurocase 11, 182–193. 10.1080/13554790590944663 16006338 PMC1307171

[B32] Nelson J. McKinley R. A. Phillips C. McIntire L. Goodyear C. Kreiner A.. (2016). The effects of transcranial direct current stimulation (tDCS) on multitasking throughput capacity. Front. Hum. Neurosci. 10:589. 10.3389/fnhum.2016.00589 27965553 PMC5126079

[B33] Nitsche M. A. Paulus W. (2001). Sustained excitability elevations induced by transcranial DC motor cortex stimulation in humans. Neurology 57, 1899–1901. 10.1212/WNL.57.10.1899 11723286

[B34] Oldfield R. C. (1971). The assessment and analysis of handedness: the Edinburgh inventory. Neuropsychologia 9, 97–113. 10.1016/0028-3932(71)90067-4 5146491

[B35] Pedersen P. M. Vinter K. Olsen T. S. (2004). Aphasia after stroke: type, severity and prognosis the Copenhagen aphasia study. Cerebrovasc. Dis. 17, 35–43. 10.1159/000073896 14530636

[B36] Peters H. T. Edwards D. J. Wortman-Jutt S. Page S. J. (2016). Moving forward by stimulating the brain: transcranial direct current stimulation in post-stroke hemiparesis. Front. Hum. Neurosci. 10:394. 10.3389/fnhum.2016.00394 27555811 PMC4977294

[B37] Polanía R. Paulus W. Antal A. Nitsche M. A. (2011). Introducing graph theory to track for neuroplastic alterations in the resting human brain: a transcranial direct current stimulation study. Neuroimage 54, 2287–2296. 10.1016/j.neuroimage.2010.09.085 20932916

[B38] Polania R. Paulus W. Nitsche M. A. (2012). Modulating cortico-striatal and thalamo-cortical functional connectivity with transcranial direct current stimulation. Hum. Brain Mapp. 33, 2499–2508. 10.1002/hbm.21380 21922602 PMC6870027

[B39] Price A. R. McAdams H. Grossman M. Hamilton R. H. (2015). A meta-analysis of transcranial direct current stimulation studies examining the reliability of effects on language measures. Brain Stimul. 8, 1093–1100. 10.1016/j.brs.2015.06.013 26210573 PMC4833093

[B40] Pulvermuller F. Neininger B. Elbert T. Mohr B. Rockstroh B. Koebbel P.. (2001). Constraint-induced therapy of chronic aphasia after stroke. Stroke 32, 1621–1626. 10.1161/01.STR.32.7.1621 11441210

[B41] Reis J. Schambra H. M. Cohen L. G. Buch E. R. Fritsch B. Zarahn E.. (2009). Noninvasive cortical stimulation enhances motor skill acquisition over multiple days through an effect on consolidation. Proc. Natl. Acad. Sci. U.S.A. 106, 1590–1595. 10.1073/pnas.0805413106 19164589 PMC2635787

[B42] Richardson J. Datta A. Dmochowski J. Parra L. C. Fridriksson J. (2015). Feasibility of using high-definition transcranial direct current stimulation (HD-tDCS) to enhance treatment outcomes in persons with aphasia. NeuroRehabilitation 36, 115–126. 10.3233/NRE-141199 25547776 PMC5764169

[B43] Robey R. R. (1998). A meta-analysis of clinical outcomes in the treatment of aphasia. J. Speech Lang. Hear. Res. 41, 172–187. 10.1044/jslhr.4101.172 9493743

[B44] Rosen H. J. Petersen S. E. Linenweber M. R. Snyder A. Z. White D. A. Chapman L.. (2000). Neural correlates of recovery from aphasia after damage to left inferior frontal cortex. Neurology 55, 1883–1894. 10.1212/WNL.55.12.1883 11134389

[B45] Russo R. Wallace D. Fitzgerald P. B. Cooper N. R. (2013). Perception of comfort during active and sham transcranial direct current stimulation: a double blind study. Brain Stimul. 6, 946–951. 10.1016/j.brs.2013.05.009 23835166

[B46] Saffran E. M. Berndt R. S. Schwartz M. F. (1989). The quantitative analysis of agrammatic production: procedure and data. Brain Lang. 37, 440–479. 10.1016/0093-934X(89)90030-8 2804622

[B47] Sarkar A. Dowker A. Cohen Kadosh R. (2014). Cognitive enhancement or cognitive cost: trait-specific outcomes of brain stimulation in the case of mathematics anxiety. J. Neurosci. 34, 16605–16610. 10.1523/JNEUROSCI.3129-14.2014 25505313 PMC4261089

[B48] Saur D. Lange R. Baumgaertner A. (2006). Dynamics of language reorganization after stroke. Brain 129, 1371–1384. 10.1093/brain/awl090 16638796

[B49] Schlaug G. Marchina S. Norton A. (2009). Evidence for plasticity in white-matter tracts of patients with chronic Broca's aphasia undergoing intense intonation-based speech therapy. Ann. N.Y. Acad. Sci. 1169, 385–394. 10.1111/j.1749-6632.2009.04587.x 19673813 PMC2777670

[B50] Sehm B. Kipping J. Schäfer A. Villringer A. Ragert P. (2013). A comparison between uni- and bilateral tDCS effects on functional connectivity of the human motor cortex. Front. Hum. Neurosci. 7:183. 10.3389/fnhum.2013.00183 23675337 PMC3646257

[B51] Shah-Basak P. P. Norise C. Garcia G. Torres J. Faseyitan O. Hamilton R. H. (2015). Individualized treatment with transcranial direct current stimulation in patients with chronic non-fluent aphasia due to stroke. Front. Hum. Neurosci. 9:201. 10.3389/fnhum.2015.00201 25954178 PMC4404833

[B52] Shah-Basak P. P. Wurzman R. Purcell J. B. Gervits F. Hamilton R. (2016). Fields or flows? A comparative metaanalysis of transcranial magnetic and direct current stimulation to treat post-stroke aphasia. Restor. Neurol. Neurosci. 34, 537–558. 10.3233/rnn-150616 27163249

[B53] Siebner H. R. Bergmann T. O. Bestmann S. Massimini M. Johansen-Berg H. Mochizuki H.. (2009). Consensus paper: combining transcranial stimulation with neuroimaging. Brain Stimul. 2, 58–80. 10.1016/j.brs.2008.11.002 20633405

[B54] Siebner H. R. Ziemann U. (2010). Rippling the cortex with high-frequency (>100 Hz) alternating current stimulation. J. Physiol. 588(Pt 24), 4851–4852. 10.1113/jphysiol.2010.200857 21173085 PMC3036180

[B55] Stagg C. J. Lin R. L. Mezue M. Segerdahl A. Kong Y. Xie J.. (2013). Widespread modulation of cerebral perfusion induced during and after transcranial direct current stimulation applied to the left dorsolateral prefrontal cortex. J. Neurosci. 33, 11425–11431. 10.1523/JNEUROSCI.3887-12.2013 23843514 PMC3724554

[B56] National Stroke Association (2008). Available online at: http://www.stroke.org

[B57] Szekely A. Jacobsen T. D'amico S. Devescovi A. Andonova E. Herron D.. (2004). A new on-line resource for psycholinguistic studies. J. Mem. Lang. 51, 247–250. 10.1016/j.jml.2004.03.002 23002322 PMC3446821

[B58] Turkeltaub P. E. Benson J. Hamilton R. H. Datta A. Bikson M. Coslett H. B. (2012). Left lateralizing transcranial direct current stimulation improves reading efficiency. Brain Stimul. 5, 201–207. 10.1016/j.brs.2011.04.002 22305346 PMC3346858

[B59] Uehara K. Coxon J. P. Byblow W. D. (2015). Transcranial direct current stimulation improves ipsilateral selective muscle activation in a frequency dependent manner. PLoS ONE 10:e0122434. 10.1371/journal.pone.0122434 25816204 PMC4376864

[B60] Wade D. T. Hewer R. L. David R. M. Enderby P. M. (1986). Aphasia after stroke: natural history and associated deficits. J. Neurol. Neurosurg. Psychiatr. 49, 11–16. 10.1136/jnnp.49.1.11 2420939 PMC1028640

[B61] Winhuisen L. Thiel A. Schumacher B. Kessler J. Rudolf J. Haupt W. F.. (2005). Role of the contralateral inferior frontal gyrus in recovery of language function in poststroke aphasia: a combined repetitive transcranial magnetic stimulation and positron emission tomography study. Stroke 36, 1759–1763. 10.1161/01.STR.0000174487.81126.ef 16020770

[B62] Wu D. Wang J. Yuan Y. (2015). Effects of transcranial direct current stimulation on naming and cortical excitability in stroke patients with aphasia. Neurosci. Lett. 589, 115–120. 10.1016/j.neulet.2015.01.045 25603474

[B63] Yi Y. G. Chun M. H. Do K. H. Sung E. J. Kwon Y. G. Kim D. Y. (2016). The effect of transcranial direct current stimulation on neglect syndrome in stroke patients. Ann. Rehabil. Med. 40, 223–229. 10.5535/arm.2016.40.2.223 27152271 PMC4855115

